# Exploring the Impact of Emotional Eating Among University Students: A Literature Review

**DOI:** 10.3390/medsci13020056

**Published:** 2025-05-05

**Authors:** Olga Alexatou, Sousana K. Papadopoulou, Maria Mentzelou, Georgia-Eirini Deligiannidou, Antonios Dakanalis, Constantinos Giaginis

**Affiliations:** 1 Department of Food Science and Nutrition, School of Environment, University of Aegean, 81100 Myrina, Greece; rd.olga.alexatou@gmail.com (O.A.); maria.mentzelou@hotmail.com (M.M.); 2 Department of Nutritional Sciences and Dietetics, School of Health Sciences, International Hellenic University, 57001 Thessaloniki, Greece; souzpapa@gmail.com (S.K.P.); deligiannidoueirini@yahoo.gr (G.-E.D.); 3 Department of Mental Health, Fondazione IRCCS San Gerardo dei Tintori, Via G.B. Pergolesi 33, 20900 Monza, Italy; antonios.dakanalis@unimib.it; 4 Department of Medicine and Surgery, University of Milan Bicocca, Via Cadore 38, 20900 Monza, Italy

**Keywords:** emotional eating, depression, anxiety, stress, social media overuse, nutritional habits, COVID-19 lockdown, university students, quality of life, academic performance

## Abstract

Background/Objectives: Emotional eating has been considered as a trend to consume energy concentrated and tasty foods in response to adverse emotions. Emotional eating may harmfully influence physical and mental health among university students, worsening their daily quality of life and their academic performance. The aim of the present study is to critically summarize and analyze the currently available clinical data concerning the impact of emotional eating among university students. Methods: Comprehensive exploration of the currently available scientific literature was performed in the most precise scientific databases, utilizing relevant and representative keywords. Results: More than a few interrelationships were found between emotional eating and body mass index, physical activity, depression, anxiety, stress, social media overuse, nutritional behaviors, and COVID-19 lockdown concerning university students. Conclusions: The currently available clinical studies support evidence that there are significant intercorrelations between emotional eating and several aspects of physical and mental health of university students. However, most of them have a cross-sectional design that cannot establish causality effects. In this respect, prospective surveys are strongly required to delineate the impact of emotional eating in the daily life of university students.

## 1. Introduction

Emotional eating has been identified as the trend of consuming energy-dense, nutrient-poor, and tasty foods like foods with enhanced sugar, fat, and energy content in response to negative emotions, containing anxiety and depressive symptoms, negative self-concept, anger, fear, dullness, melancholy, and loneliness, as a means of coping with emotions’ fluctuations, etc. [1]. Emotional eating is also considered as eating in response to an emotional situation rather than hunger, frequently as a manner of emotional comfort or as a method to manage harmful emotions in unhealthy habits [2]. Emotional eating has been recognized as the overlapping mechanism between reward circuitry, cognitive control, and emotions. Certain pathophysiological mechanisms like an energy imbalance in the hypothalamus and its relations to ghrelin (the “hunger hormone”) and leptin (the “body’s satiety signal”) exhibit a substantial effect on mood illnesses [3,4]. Either overeating is induced by emotional eating or, conversely, emotional eating as a coping practice for the sadness related to being overweight may result in an enhanced BMI. In view of the above evidence, it was shown that overweight individuals preferred to consume more foods that make them feel terrible, while underweight individuals remarkably tend to consume more foods, which makes them feel pleasant. Thus, individuals that are disappointed with their body may be more probable to use foods as a coping mechanism [3,4,5].

Furthermore, emotional eating has appeared as a persistent behavioral phenomenon associated with obesity and obesity-related cardiometabolic diseases like diabetes, hypertension, and hyperlipidemia [3,4]. In addition, several studies have supported evidence that overeating/obesity and no healthy nutritional habits (e.g., fast-food intake) are related to emotional eating [5]. In this aspect, the effect of nutritional consumption on physical health has been widely investigated; however, novel investigations have suggested that nutritional habits may similarly affect or may be influenced by mental health [6,7]. Hence, investigating nutritional behaviors such as emotional eating could offer novel insights across a broad range of probable health outcomes. In addition, COVID-19 lockdown increased the probability of depression, anxiety, and stress, which, in turn, increased the prevalence of emotional eating [8,9]. Most of the above evidence generally concerns the effect of emotional eating in the general population and especially in adults. However, while COVID-19 had psychological effects, not all populations were affected equally. In this respect, several differences in age, sex, socioeconomic status, or cultural factors may be mediated between the relationship of the COVID-19 pandemic with mental health disorders, including emotional eating.

In the recent two decades, there has been an increasing incidence of people suffering from emotional eating. Notably, a recent meta-analysis study including 18 clinical surveys and 21,237 individuals reported a 44.9% (95% CI: 29–62%) prevalence of emotional eating [10]. However, heterogeneity was found to be high, which may be ascribed to the difference in measurement tools used for emotional eating and differences in weight status or geographical regions [10]. To investigate emotional eating, a tool is required to determine it. In this respect, various self-reported questionnaires on emotional eating have been intended. Among the most utilized tools are the Three-Factor Eating Questionnaire (TFEQ), the Dutch Eating Behavior Questionnaire (DEBQ), and the Emotional Eater Questionnaire (EEQ), which differ in their conceptualization of emotional eating, the range of emotions assessed, and their scoring systems [11,12,13]. TFEQ exhibited good validity and reliability for determining eating behavior in people affected by obesity, providing a multidimensional approach by effectively differentiating among diverse dietary habits concerning the general population [14,15]. The advantages of the DEBQ are its multidimensional methodology, assessing three diverse characteristics of eating behavior (emotional, external, and restrictive). It also provides a more thorough picture of an individual’s eating behavior [16,17]. EEQ is a reliable and valid tool for measuring emotional eating in overweight adults and could be a relevant tool against obesity, which can be proved useful for identifying individuals who consume food in response to emotions, distinguishing them from other forms of eating behavior [18,19].

The university period seems to enhance the probability of diverse unhealthy habits, such as physical inactivity, increased junk food intake, evening snacking, enhanced perceived stress levels, elevated workload, nutritional restraint, living in residence halls, and alcohol consumption [20,21,22]. The transition period from adolescence to adulthood at the initial years of university studies constitutes a crucial interval for young adults, who are introduced to a new social environment and a different educational system with increased daily demands. In this aspect, university students may exhibit a greater probability of developing depression, anxiety, stress, anger, and sleep disturbance symptoms, restrictive, emotional, and external eating behaviors, as well as diverse psychopathological states like mood illnesses, perceived stress, and attention-deficit/hyperactivity disorder [23,24,25,26]. Substantial evidence also highlighted the harmful effect of COVID-19 lockdown on the mental health of university students, especially in association with anxiety, depression, and stress symptomatology [27,28]. Unhealthy dietary behaviors like a decreased Mediterranean diet compliance may also enhance the probability of depression, anxiety, and stress concerning university students [29]. Internet, smartphone addiction, gaming disorders, low self-esteem, loneliness, sleep disturbances, suicidal ideations, eating illnesses, and alcohol and drug addiction are also common in university students’ populations [30]. An inverse association was also found between social media addiction and students’ academic performance [31]. Moreover, university students constitute a distinct population group showing alterations in BMI values over the following educational years, with an upward tendency for BMI [32]. All the above factors may increase the risk of developing emotional eating, which may negatively affect students’ mental well-being and academic performance. On the other hand, unhealthy eating behaviors exhibit also a negative impact on students’ academic performance [33]. Thus, university students aged 8–25 years old are highly vulnerable to developing eating disturbances such as emotional eating.

Based on the above considerations, the current survey aimed to provide for the first time a thorough literature review of the currently existing studies analyzing the reported interrelationships among emotional eating and sociodemographic and anthropometric parameters, lifestyle factors, and dietary habits.

Comprehensive investigation of the currently available international literature was conducted on the most accurate scientific databases, e.g., PubMed, Scopus, Web of Science, and Google Scholar, utilizing relevant and characteristic keywords like emotional eating, overeating, psychological disorders, eating behavior, binge eating, depression, anxiety, stress, obesity, overweight, weight gain, body mass index (BMI), appetite, hunger, university students, college students, young adults, etc. We only included longitudinal, cross-sectional, and descriptive clinical surveys with Caucasian individuals. Only surveys written in the English language were included. Review articles, case report studies, commentaries, and abstracts in congress proceedings as well as animal studies were excluded. The findings were chosen based on relevance and the most appropriate ones were opted for and reported below. Clinical human surveys with an adequate protocol design which used a validated questionnaire to identify emotional eating were included in this review. The retrieved surveys were additionally reviewed for relevant surveys, which were referred to their manuscript. The recovered surveys were similarly cautiously checked for relevant surveys cited in their manuscript. No exclusion criteria were utilized regarding the period of publication of the recovered clinical surveys.

**Figure 1:**
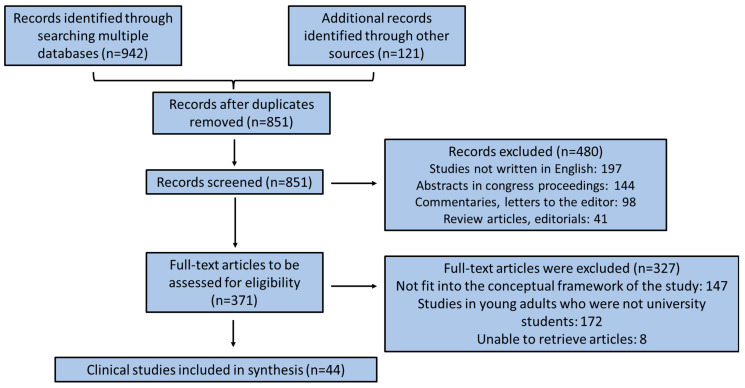
Flow chart diagram for clinical studies’ enrollment.

All authors worked as reviewers. To enhance reliability, all authors collaborated in pairs and critically reviewed all recovered research articles, analyzed their results, and adjusted the data extraction manually prior to beginning the screening for this review. They alternately evaluated the titles, abstracts, and then the full manuscripts of all publications recovered from their research for possibly related publications with adequate methodology and reliable survey design. A data charting form was established along with reviewers (O.A. and C.G.), who independently recorded the data and scrutinized the results and the charting format in an iterative procedure. In Figure 1, a flow chart diagram is presented illustrating the selection of relevant clinical surveys.

## 2. Results

A comprehensive search of the current scientific literature was performed, which identified 44 clinical studies exploring the impact of emotional eating among university students. All clinical studies are included in Table 1 [34,35,36,37,38,39,40,41,42,43,44,45,46,47,48,49,50,51,52,53,54,55,56,57,58,59,60,61,62,63,64,65,66,67,68,69,70,71,72,73,74,75,76,77]. More to the point, a cross-sectional descriptive study was carried out to evaluate the influence of sociodemographic factors of 537 Turkish university students on emotional eating behavior assessed by Emotional Eating Scale (EES) [34]. This survey showed a positive association of emotional eating with BMI and body weight. Moreover, certain variables like will, anger, BMI, and body weight were identified predictor factors of emotional eating [34]. This survey supported evidence that emotional eating could be a coping response to harmful emotions. It also implied that negative emotions may be the most crucial factor influencing emotional eating. Hence, it was speculated that the psychological dimensions of incorrect eating habits need to be addressed [34].

Another cross-sectional survey aimed to explore the impact of eating behavior measured by DEBQ in 548 Japanese university underweight and normal-weight students [35]. In total, 38.6% of students were classified as emotional or very emotional eaters. An association was found between BMI and emotional eating [35]. Specifically, students with obesity had a higher risk of being very emotional eaters (9.8%) compared to overweight (4.4%) or normal-weight (7.2%) students. Amongst obese students, women showed higher levels of emotional eating than men (48.3% vs. 25%, respectively). Moreover, emotional eating was weakly, positively associated with BMI in female students [35].

Amoako et al. also performed a cross-sectional survey exploring emotional eating, uncontrolled eating, and restrained eating behaviors assessed by TFEQ in 129 university students in Ghana [36]. Among the three dimensions, emotional eating was the only one associated with any of the health outcomes in this survey, such as BMI concerning males and anxiety and sleep quality regarding females. Females affected by obesity and overweight reported significantly elevated emotional eating levels than normal-weight females. No such results were noted amongst males affected by obesity or overweight [36]. In addition, emotional eating, uncontrolled eating, and restrained eating levels were not different amongst males and females. Although this survey provided evidence for the eating behaviors of Ghanaian university students and permitted the comparison of the students from other cultures, additional work should be developed by culturally relevant tools for the Ghanaian population [36].

A longitudinal study applied a person-oriented approach and latent class analysis to investigate the negative emotional eating patterns assessed by Adult Eating Behavior Questionnaire (AEBQ) and to characterize these patterns in a sample of 1068 Chinese university students [37]. Four patterns, namely, “non-emotional eating” (38.9%), “emotional over- and under-eating” (15.4%), “emotional over-eating” (14.7%), and “emotional under-eating” (31.0%), were recognized. Both gender and BMI were identified as crucial predictive factors for harmful emotional eating behaviors [37]. The detected four patterns revealed significant differences in eating disease symptomatology and psychological distress. Specifically, participants in emotional over- and under-eating presented the greatest levels of eating disorder symptomatology and psychological distress. Collectively, this survey recognized four distinct harmful emotional eating behaviors, amongst which emotional over- and under-eating was the most difficult [37].

A randomized control experimental study was directed to evaluate the impact of internet-based solution-related short-term group psychotherapy on emotional eating levels assessed by DEBQ in 55 Turkish nursing university students [38]. Sixty students were enrolled in experimental and control groups by simple random sampling methodology. Those in the experimental group were subjected to solution-related psychotherapy, and those in the control group were subjected to healthy nutrition training. A substantial difference was found amongst the pre-test, post-test, and follow-up test BMI of those in the experimental group [38]. In addition, a considerable difference was found amongst the group’s pre- and post-test emotional eating levels, whereas no significant difference was found between their Difficulties in Emotion Regulation Scale scores. Thus, the short-term solution-focused approach was found to be effective in reducing the emotional eating levels of university students [38].

A longitudinal study was designed to assess gender differences in stress, emotional eating, trend of overeating assessed by TEFQ, and restrained eating behavior in 173 USA university freshmen [39]. In gender-adjusted linear regression modeling, positive associations between baseline TFEQ emotional eating sub-scales and baseline weight BMI, waist circumference, and fat mass index were noted. Based on questionnaire scores, university freshmen showed a higher trend in overeating in response to external cues and emotions tended to exhibit higher weight, BMI, and waist circumference at the beginning of university studies [39]. Males presenting greater perceived stress at university entrance afterwards presented considerably higher body weight in the first semester. The above association was not found in female students. Collectively, psychological constructs, including eating competence, restrained eating, disinhibited eating, emotional eating, and perceived stress, were related with anthropometric parameters and adiposity in university students at the first academic year [39].

Even though emotional eating and personality traits have been recognized as crucial predictive factors of eating disorders, their impact in obesity in the absence of eating diseases has not been elucidated yet. In this aspect, a cross-sectional survey was designed to investigate the cumulative effect of emotional eating evaluated by Emotional Overeating Questionnaire (EOQ) and personality traits on overeating behavior in 266 Italian university students classified according to their BMI [40]. This study showed a psychologic relationship of rising overeating behavior and decreased self-directedness in combination with greater sadness and anger. Nonetheless, students without eating disorders may overeat independently of this emotional/personological configuration [40]. Moreover, this study found that overweight and/or obese students in the absence of eating diseases exhibited more binge episodes in comparison with overweight and normal-weight students, also eliminating the cumulative impact of emotional antecedents and personality traits [40].

A cross-sectional survey was performed including 353 Polish university students to evaluate the relationships between eating behavior assessed by TFEQ and age, socioeconomic status, physical activity, BMI, waist-to-height ratio (WHtR), and social desirability [41]. Emotional eating was positively correlated with BMI among females. Social desirability was negatively associated with uncontrolled eating and emotional eating among females. Multivariate analysis indicated that, among males, physical activity exerted a main effect on emotional eating [41]. Among females, positive associations of cognitive restraint with physical activity and BMI were noted. Moreover, a positive association of emotional eating with BMI was recorded. Social desirability exhibited the greatest main effect on eating behavior among females, being inversely related to uncontrolled eating and emotional eating. Thus, the above survey provided evidence that physical activity, BMI, WHtR, and social desirability were related to self-reported eating behavior in university students [41].

Shatwan et al. performed another cross-sectional study designed to investigate if sociodemographic parameters and health-related factors, containing BMI and physical activity, could be related to perceived stress, emotional eating determined by EEQ, and healthy eating index in 434 Saoudi Arabia university students [42]. Emotional eating was considerably associated with short healthy eating index score, fruit juice, fruit intake, additional sugar, and saturated fatty acids [42]. Academic major was related to perceived stress and emotional eating. Moreover, high BMI was related to high emotional eating levels. In fact, obesity and being overweight were considerably associated with emotional eating and body weight reduction exerted a positive impact on emotional eating [42].

Another cross-sectional study explored the emotional patterns evaluated by Adult Eating Behavior Questionnaire (AEBQ) of 553 American college students, which was a repetition of the survey performed by He et al. [37] on Chinese college students [43]. A solution with four groups was developed, i.e., emotional over- and undereating (18.3%), emotional overeating (18.2%), emotional undereating (27.8%), and non-emotional eating (35.7%). The above results repeated and extended the previous results by He et al. [37] concerning emotional over- and under-eating class having the greatest probability of depression, anxiety, stress, and psychosocial damage because of eating disorder symptomatology and poorer psychological flexibility [43]. University students having troubles with awareness and acknowledgement of their emotions appeared to retain the most difficult form of emotional eating and may provide assistance from dialectical behavioral therapy and acceptance and commitment to therapeutic skills education [43].

Shatwant et al. designed a cross-sectional survey to evaluate the incidence of abnormal eating behaviors, social media use, and any association of social media use with eating behaviors in 272 medical university students [44]. Two thirds of the students (74.4%) participated in at least three social media platforms and over a quarter of students (26.10%) participated in four or more social media platforms. In total, 22.1% of the enrolled students had disordered eating behaviors. A positive correlation was found among TFEQ domains like uncontrolled eating, emotional eating, and TFEQ total score with the Scale of Effects of Social Media on Eating Behavior scores [44]. Thus, the above survey found a considerable association of elevated social media use with developing disordered eating behaviors in medical students, highlighting the necessity for the development of strategies modulating social media use with eating behaviors in mind [44].

A cross-sectional descriptive survey was designed to explore emotional eating in 575 college students in Israel, in particular, during the period of war, which describes rare and heightened stressors that gather on top [45]. Most students were Jewish (80%), and 42% stayed in the conflict zone. This study indicated that factors like female gender, not having children, earlier age, elevated BMI, and enhanced stress were predictive factors of heightened emotional eating [45]. Moreover, age and body satisfaction were significantly, moderately associated with emotional eating, revealing that younger students and those with reduced body satisfaction stated greater levels of emotional eating. Stress and daily social media use both exhibited significant, moderate, and positive correlations with emotional eating. This implied that students experiencing higher stress and spending more time on social media reported engaging in emotional eating more frequently [45].

Another cross-sectional study aimed to explore the eating behavior assessed by TFEQ and lifestyle of 227 healthcare university students in Poland [46]. BMI, depression, and impulsiveness levels were greater in students presenting an elevated emotional eating score. Moreover, emotional eaters showed elevated levels concerning cognitive restraint and uncontrolled eating domains. Higher intake of sugar, sweets, and snacks in students with elevated emotional eating scores was observed [46]. Elevated scores of specific eating behaviors were associated with body weight, compliance to the Mediterranean diet, and intake of specific product groups (sweets and alcohol). The findings of the above survey may contribute to a deeper estimation of the nutritional habits and overweight/obesity in university students, supporting the development of strategies to encourage healthy lifestyles in that population [46].

Chearskul et al. showed that the TFEQ Thai version had adequate internal reliability and test–retest consistencies in 89 Thai medical students [47]. Restraint and disinhibition levels that were elevated in females compared to males were associated with body fat content but were not associated with BMI. Disinhibition differed clearly with restraint and hunger, whereas restraint was inversely related to hunger [47]. Reduced restraint was noted in students who stated liking carbohydrate, which is a main daily macronutrient in the Thai population, whereas those who stated liking dietary fiber had the greatest restraint scores and minimal hunger scores [47].

A cross-sectional study intended to explore the relationships amongst emotional eating, perceived stress, contextualized stress, and BMI in 99 African American female university students [48]. Eating behavior was evaluated with the Revised Eating Behavior Pattern Questionnaire’s (REBPQ) subscale for Emotional Eating. This study found that perceived stress was associated with emotional eating, whereas contextualized stress was not associated with emotional eating [48]. Moreover, BMI was not considerably associated with perceived stress, contextualized stress, or emotional eating. On the other hand, a substantial eating behavior patterns × perceived stress interaction was noted concerning BMI. Accordingly, a substantial eating behavior patterns × contextualized stress interaction was evident regarding BMI. Thus, this study demonstrated that stress experience may interact with emotional eating to affect BMI [48].

Liu et al. performed a cross-sectional study to investigate potential relationships of emotional eating assessed by TFEQ with depression and laryngopharyngeal reflux in 1301 Chinese college students [49]. Elevated emotional eating was stated by 52.7% of students. Female students had higher emotional eating scores compared to male students. Moreover, university students whose major was liberal art, with elevated BMI, and with high physical inactivity levels exhibited increased emotional eating levels [49]. Among those with depressive symptomatology, 67.8% exhibited elevated emotional eating levels, which was considerably increased in comparison to those not presenting depression symptomatology (49.2%). The incidence of depression symptomatology was 18.6% and that of laryngopharyngeal reflux symptoms was 8.1%. Both emotional eating and depressive disease severity was related to laryngopharyngeal reflux symptomatology [49].

Another cross-sectional study intended to explore early-risk alcohol drinking and alcohol addiction, Mediterranean diet compliance, and emotional eating assessed by Emotional Eater Questionnaire (EEQ) in 584 Spanish university students [50]. Overall, 63.6% of students exhibited reduced compliance to the Mediterranean diet, with no differences by gender. Based on the Alcohol Use Disorders Identification Test questionnaire, 26.2% of students were classified as high-risk drinkers and 7.7% as alcohol-addicted [50]. About 38.6% of the students were categorized as eating very emotionally or eating emotionally, and 37.2% were categorized as low emotional eaters. EEQ was positively but weakly associated with BMI in female students. Hence, university students showed a reduced compliance to the Mediterranean diet, significant high-risk alcohol drinking, and decreased emotional eating [50].

Wang et al. designed a cross-sectional survey to investigate possible associations among physical activity, self-identity, social anxiety, and emotional eating in 373 overweight and obese university students affected by overweight or obesity [51]. Emotional eating was assessed utilizing four items of the Emotional Eating Scale. This study revealed that physical activity considerably influenced self-identity and social anxiety that subsequently impacted emotional eating at a significant level. In addition, self-identity and social anxiety served as mediators in the association of physical activity with emotional eating. The above findings emphasized the impact of physical activity in mitigating emotional eating in overweight or obese university students [51].

Accordingly, a cross-sectional survey was performed to examine the physical activities and nutritional habits of 1000 university students in Korea regarding their perceived stress levels [52]. Among other findings of this study, female students were found to tend to eat to release stress as a way to reduce this kind of mood. It was found that female students ate more frequently to release stress and consumed more than two pieces of fresh fruits compared to males [52]. No healthy nutritional habits were more widespread in females, as they more frequently consumed snacks like cakes, candies, and soft drinks and skipped dinner compared to males. University students presented two different behaviors during stress conditions, including the more frequent consumption of specific foods (overeating) and less frequent eating like skipping meals (undereating) [52]. However, it should be noted that this study did not use any validated questionnaire for assessing emotional eating.

Another cross-sectional study aimed to examine the incidence and factors associated with emotional eating determined by DEBQ in 424 university students [53]. This survey found that there was more than a 3-fold higher probability of negative emotional eating in females (14.8%) in comparison with male students (4.5%). Presenting at least low depression symptomatology was the only independent factor related to negative emotional eating in males [53]. Concerning females, negative emotional eating was independently related to not having a romantic partner, presenting depression symptoms, and exhibiting at least mild stress. Anxiety levels were not independently related to harmful emotional eating for both genders. Both male and female students with harmful emotional eating exhibited considerably decreased self-perceived health levels, increased BMI, and reduced life satisfaction levels [53].

Zhou et al. carried out a cross-sectional survey to investigate the mediating effect of depression and the moderating effect of physical activity in the association of sleep quality with emotional eating assessed by DEBQ in 813 university students [54]. After adjusting for gender, age, and BMI, sleep quality was independently associated with emotional eating. Depression mainly mediated the above relation between them. Moreover, physical activity diminished the association of sleep quality with emotional eating through depression [54]. Depression substantially predicted emotional eating in students with low physical activity, but it was not significant concerning students presenting moderate or high physical activity. This study implied the importance of presenting more sleep hygiene learning and physical activity in university settings [54].

A longitudinal study aimed to investigate under what conditions and mechanisms stress could be related to emotional eating among 232 university students in the USA [55]. The Emotion and Stress-Related Eating subscale of the Eating and Appraisal Due to Emotions and Stress Questionnaire (EADES) was used to assess the extent to which students use food to cope with emotions and/or stressors. At baseline, emotional eating was significantly associated with perceived stress, barriers to and motivators of healthy eating, and avoidance coping but not approach coping [55]. Furthermore, avoidance coping facilitated and moderated the association of perceived stress with emotional eating. However, baseline stress levels were not related to emotional eating one year afterwards. This study supported substantial evidence that university students utilizing avoidance coping approaches could be especially vulnerable to the impacts of stress on emotional eating [55].

A pilot cross-sectional survey was performed to investigate the relationship among the tendency towards emotional eating assessed by EEQ and multiple health-related behaviors like extreme internet use or elevated alcohol drinking in 56 Spanish university students [56]. Additionally, this study aimed to examine the relationship of the above risky behaviors with the students’ performance level in a virtual reality task, which assessed their superior functioning, and to evaluate impulsivity and anxiety and depression symptoms’ severity [56]. This study showed an association of emotional eating with extreme internet use but not with alcohol drinking. Emotional eating and internet overuse were not associated with superior function but were related to impulsivity, depression, and anxiety. Impulsivity and depression symptoms were responsible for 45% of the variance in emotional eating [56].

Another cross-sectional survey designed to evaluate the interrelationships amongst perceived stress, eating self-regulation, emotional eating, and food consumption in 523 undergraduate university students in USA [57]. Emotional eating was determined by the 25-item Emotional Eating Scale. Perceived stress was positively related to emotional eating and negatively related to eating self-regulation [57]. Eating self-regulation partly mediated the relation of perceived stress with emotional eating. Positive associations of emotional eating with the consumption of sweets and soft drinks were noted. Eating self-regulation was inversely associated with sweet consumption. Eating self-regulation and emotional eating wholly mediated the relation of perceived stress with sweet consumption [57].

An exploratory qualitative study was carried out, involving 3-day food journals and in-depth interviews, with proportional measure sampling of eight male and eight female university students [58]. This study aimed to obtain an insight into students’ experiences of their emotional eating attitudes assessed by Weight Related Eating Questionnaire (WREQ). This study found gender-related differences and similarities [58]. Stress was recognized as the major stimuli for emotional eating concerning female students, frequently followed by guilt. Unpleasant feelings like boredom or anxiety turning to food as a distraction were recognized as the major stimuli concerning male students. Nevertheless, male students were less probable to experience guilt next to an emotional eating episode compared to female students. Throughout emotional eating experiences, both genders selected what they identified as no healthful foods [58].

Constant et al. carried out a cross-sectional survey to characterize emotional overeating in normal-weight university students and especially in 377 female students in France [59]. Emotional overeating was assessed by the Emotional Overeating Questionnaire (EOQ) evaluating eating in response to six emotions, specifically anxiety, sadness, loneliness, anger, fatigue, and happiness. TFEQ was used to measure cognitive and behavioral domains of eating. This survey reported that half of the students adopted intermittent emotional overeating in the previous 28 days, mainly throughout 1 to 5 days in the previous 28 days, in response to anxiety (51.3%), loneliness (45.1%), sadness (44.8%), and happiness (43.6%) and, to a lower degree, in response to tiredness (27.4%) and anger (14.6%). In multivariate analysis, distress-stimulated overeating was positively associated with inability to refuse emotional cues, disordered eating symptoms, and failure of control over food consumption. It was also negatively associated with moderate and excessive drinking [59].

An exploratory qualitative study conducted on 43 female university students found that self-reported levels of emotional eating assessed by TFEQ predicted food intake following stress only for those participants demonstrating high stress reactivity and greater emotional relief from eating [60]. The above survey demonstrated that self-assessment of emotional eating in the aggregate was not regularly related to elevated food consumption in response to stress or harmful emotions [60]. The above finding was ascribed to the relationship between self-reported emotional eating and behavioral measures of emotional eating was attenuated by the stress response and emotional relief of stress by eating. The above survey also underscored the proposal that diverse features may be able to mitigate or increase the impacts of emotional eating on eating behaviors [60].

A cross-sectional study aimed to investigate the associations of eating behavior dimensions with psychopathological symptoms in 258 Portuguese undergraduate students [61]. Emotional and external eating were determined by DEBQ. In both the female and male students, the psychopathological symptoms were positively associated with emotional and binge eating. Moreover, positive associations of psychopathological symptoms and external eating in female students were noted, whereas negative associations with eating self-efficacy in male students were recorded [61]. Multivariate analysis indicated that most eating behavior dimensions (all except external eating and flexible control in male students) were greatly ascribed to BMI and psychopathology [61].

Another cross-sectional survey was designed to investigate the mediating effect of emotional regulation complications in the associations among worries of compassion and emotional eating evaluated by DEBQ in 673 Chinese university students, as well as the sex difference in the mediation model [62]. This study indicated that both worries of compassion for self and worry of compassion from others were positively related to emotional regulation troubles, which were associated with emotional eating concerning female students. Emotional regulation complications exerted a considerable mediating impact in the association of worries of compassion with emotional eating [62]. Relatively, for male students, merely worry of compassion for self was positively related to emotional regulation complications. However, emotional regulation complications were not associated with emotional eating. Furthermore, the mediating impact of emotional regulation complications was not considerable in the association of worries of compassion and emotional eating for male students. The above results supported evidence that it could be crucial to improve students’ worries of compassion to decrease emotional eating, mostly for female university students [62].

Grajek et al. also carried out a cross-sectional survey in 300 Polish university students to explore and analyze the incidence of emotional eating assessed by TFEQ of students’ health-related and non-health-related fields concerning their differential health behaviors—diet and physical activity levels [63]. Emotional eating was quite frequent in 106 students (37.9%), with the non-health-related fields group students dominating (61.6%,). Elevated perceived stress levels were more often in students exhibiting emotional eating [63]. According to TFEQ, among 120 university students, behaviors similar with restricting food consumption were recorded, while 64 students showed a lack of control over food consumption. This study showed that university students having increased BMI values, no healthy nutritional habits, increased physical inactivity levels, who underestimated meal size concerning body weight and the calories, and had enhanced stress emotions were more probable to suffer from emotional eating [63].

A cross-sectional survey targeted to explore the association of eating behavior modifications with the circadian rhythm in 850 Turkish university students [64]. According to the MEQ tool, 77.1% of the students exhibited moderate circadian type classification, 15.1% were morning type, and 7.8% were evening type. It was found that, when students’ BMI values elevated, there was a reduction of 25.6% in the TFEQ levels, independently of sex, and a rise of 10.6% in their chronotype scores, which means that the students tended to be morning types. A significantly negative, very weak correlation was found between the students’ TFEQ and morningness–eveningness questionnaire (MEQ) scores. The students’ BMI and TFEQ scores were considerably affected by their MEQ scores. This survey also showed that each 1-point rise in the MEQ score resulted in a 4.0% elevation in the BMI values and a 15.8% reduction in the TFEQ levels [64].

Another survey explored social jet lag and eating behaviors in 372 university students in the USA [65]. This cross-sectional study indicated that social jet lag considerably predicted reduced intuitive eating and greater emotional eating assessed by TFEQ after adjusting for age, gender, and chronotype. It was slightly predictive of failure of managing overeating [65]. In addition, sleep quantity during weekdays (not weekends) considerably predicted intuitive eating and failure of managing overeating. Moreover, sleep quality substantially predicted intuitive eating, emotional eating, and loss of control over eating. In addition, gender was crucial for intuitive eating and emotional eating, with female students being less probable to eat spontaneously and more probable to adopt emotional eating compared to male students [65].

A cross-sectional study including 567 female Russian university students targeted to assess the potential relationships of diurnal preference with restricted eating behaviors [66]. This study showed that any of the three attributes of unhealthy eating behaviors assessed by TFEQ (i.e., absence of cognitive eating restriction, no controlled eating, and emotional eating) was connected to one or more domains of students’ chronobiological changes. Such an association was recorded for two (morning and evening) subscales of the MEQ. Cognitive eating restrictions and no controlled eating were related to the morning subscale, whereas emotional eating was associated with the evening subscale [66]. The above associations were confirmed by the relationships found for morning vs. evening aspects of earliness–lateness evaluated with two other questionnaires, (e.g., morning lateness and sleep offset vs. evening lateness and sleep onset, respectively). Thus, no healthy eating attitudes appear to be correlated with no healthy sleep–wake behaviors and to the lack of ability to wake or sleep on demand at specific times during the day [66].

Carlos et al. analyzed the Mediterranean diet compliance assessed by KIDMED questionnaire, emotional eating evaluated by EEQ, alcohol drinking, and anxiety in 252 Spanish university students and the relationships of the above covariates [67]. This cross-sectional survey showed low levels of Mediterranean diet compliance in university students (15.5%) as well as increased levels of emotional eating (29%) and anxiety (23.6%). On the other hand, alcohol addiction was reduced (2.4%). State-anxiety was a predictor of the emotional eater score and its subscales, and sex also was recognized as a predictor of subscale guilt and the overall emotional eating levels [67]. Nonetheless, Mediterranean diet compliance was only predicted by trait-anxiety. The above models accounted for 1.9% to 19%. This study also suggested the necessity for the implementation of learning settings to encourage healthful behaviors concerning the university students at risk [67].

A cross-sectional study investigated several aspects of emotional eating and their relationships with nutritional habits of 380 female students [68]. This study revealed that fat consumption was a crucial predictive factor of emotional eating and emotions of excitement. It was also found that fat consumption and educational status were considerably related to emotional eating, assessed by EES, and they could be significant predictors of emotional eating [68]. These findings highlighted the crucial role of emotional eating, its association with the intake of foods which include fat and understanding of how it happens by leading to knowledge of the significance of healthy foods for a healthy lifestyle. The above data may predict some metabolic syndromes associated with the modifying of eating disorders, contributing also to the establishment of a database of young women’s nutritional patterns and foods, principally in Saudi Arabia or the Gulf countries [68].

Another cross-sectional study determined the relationship between personality traits and dietary habits among 400 university students in Ghana [69]. Significant differences were observed between the males and females in the areas of emotional eating assessed by Emotional Eating Scale, pickiness, neophagia, fiber intake, and sugar and salt moderation. A considerable higher percentage of female compared to male students reported to be emotional eaters, picky eaters, attempting neophagia, and consuming fiber-rich foods [69]. The percentage of male students reporting intermediate sugar consumption was considerably elevated compared to the percentage of female students. Moreover, this study found that fat consumption was a considerable predictive factor of emotional eating as well as feelings of enthusiasm. Moreover, fat intake was significantly associated with educational level, and both of them could be significant predictors of emotional eating [69].

Elkin et al. designed a cross-sectional survey to explore the impact of watching mukbang on eating behavior and to underline its significance in 483 university students in Türkiye [70]. This study revealed that emotional eating, assessed by Emotional Eating Disorder Scale, was positively associated with mukbang dependence and problematic internet usage. Emotional eating was also positively related to mukbang dependence [70]. Notably, mukbang dependence exhibited a partial mediator role in the effect of problematic internet usage on emotional eating. Therefore, it was suggested that, when conducting a survey concerning emotional eating and problematic internet usage, it could be useful to investigate the incidence of mukbang watching behavior in university students [70].

A follow-up pilot survey was designed to evaluate the incidence of academic burnout and its relationship with eating disorders in 132 university students in Malaysia for an interval of one year [71]. Initially, 25.0% of the students had medium academic burnout levels, while 63.6% exhibited elevated academic burnout levels. The incidence of academic burnout next to 6–8 weeks was found to be 17.4% and 73.5% for medium and elevated levels of academic burnout, respectively [71]. Emotional eating levels assessed by TFEQ were considerably different over levels of academic burnout next to 6–8 weeks, whereas no substantial differences were noted in other subscales like cognitive restraint and uncontrolled subscales. The above results supported evidence for partial relationships of academic burnout with eating disorders [71].

A more recent study was performed to increase the knowledge of food insecurity in 232 university students in the USA [72]. In fact, this study aimed to validate the elevated incidence of foods insecurity in university student samples, examining the food insecurity–emotional eating (assessed by Emotional Eating Scale) relationship, and determining if students’ gender eating can moderate the above relationship [72]. Of the participants, 37.5% stated food insecurity. Moreover, food insecurity was positively related to emotional eating, adjusting for BMI. The above relationship was higher for male compared to female students [72].

A cross-sectional survey was directed to explore emotional and non-emotional eating habits assessed by EEQ throughout the second wave of COVID-19 in 161 female health sciences university students in Spain [73]. This survey found that emotional eaters compared to non-emotional eaters exhibited a slight food dependence and a considerably increased consumption of carbohydrates, fat, and alcohol. Moreover, energy intake was dependent on saturated fatty acids [73]. In addition, emotional eaters were not aware of their intake of calories, exhibited inadequate sleep quality, elevated perceived stress, and had low healthy eating index because of the consumption of sweets and soft drinks. The relationship between emotional eating and the consumption of sweet foods and alcoholic drinks appeared to be a persistent condition of COVID-19 pandemia [73].

Another cross-sectional survey was performed to investigate the relationship of the emotional effect of COVID-19 and emotional eating with the probability of alcohol use disorder in 456 Peruvian health sciences university students [74]. Concerning the evaluations of mental health, the Emotional Impact Profile questionnaire was used to determine the emotional impact of the COVID-19 pandemic. Mindful Eating Questionnaire (MEQ) was used to evaluate emotional eating, which characterizes a nonjudgmental awareness of the physical and feelings related to eating [74]. In total, 68.4% of the participants were emotional eaters and 8.6% stated decreased risk levels of alcohol use disorder. According to the analysis of the above study, the total emotional effect of COVID-19, overweight and obesity, depression and anxiety symptoms, and living with only one parent were identified as considerable agents, which can be related to emotional eating [74].

Furthermore, Constant et al. carried out a cross-sectional survey to estimate the proportion of 302 French female university students stating overeating in response to emotions throughout the COVID-19 university closings and to explore social and psychological agents related to this response to stress [75]. Emotional overeating was assessed using the Emotional Overeating Questionnaire (EOQ) evaluating eating in response to six feelings (anxiety, sadness, loneliness, anger, fatigue, and happiness). Nine in ten participants stated intermittent emotional overeating in the previous 28 days, mainly from the 6th to 12th day, in response to anxiety (75.5%), sadness (64.5%), happiness (59.9%), loneliness (57.9%), tiredness (51.7%), and, to a lower degree, to anger (31.1%) [75]. Exploratory factor analyses showed a one-factor latent variable representing “Distress-Induced Overeating” positively associated with internal boredom proneness, smoking, attentional impulsivity, inability to refuse emotional cues, and absence of control over food consumption and inversely with age and well-being. On the other hand, emotional overeating was not related to BMI or substance abuse [75].

Another cross-sectional survey targeted to investigate the impacts of emotional eating and social media on dietary habits and obesity in 1000 university students participating in distance education throughout the COVID-19 lockdown in Türkiye [76]. Throughout the distance education interval, more than 50% of male and female students (61.7%; 58.2%) altered their meal pattern, 31.7% of them began to intake their basic meals more systematically, and 31.2% of them skipped their basic meals. Among the enrolled students, 52.6% used social media more than 2 h daily [76]. The female students who used social media more than 2 h daily exhibited elevated levels concerning the Scale of Effects of Social Media on Eating Behavior and Emotional Eating Scale compared to those who used 2 h or less daily. The score of the Scale of Effects of Social Media on Eating Behavior was positively slightly related to BMI and positively marginally related to the emotional eating scale score. The interaction of the Scale of Effects of Social Media on Eating Behavior with emotional eating scores enhanced the probability of overweight/obesity. Collectively, concerning the students participating in distance education, social media influenced eating behavior, BMI, and emotional eating. Furthermore, the above impacts could enhance the likelihood of overweight/obesity [76].

Shehata et al. performed another cross-sectional survey in 580 people, including medical sciences students, during an interval of one month at the last part of COVID-19 lockdown [77]. Emotional eating was assessed by Emotional Eating Scale. More than two fifths of students, employees, and staff (45.2%, 45.5%, and 44.2%, respectively) reported that their body weight elevated due to the lockdown. In addition, 50.8% of students, 42.5% of employees, and 54.6% of staff showed medium emotional eating scores [77]. Likewise, the majority of students, employees, and staff showed medium levels of stress (84%, 80.8%, and 76.1%, respectively). The overall emotional eating levels were positively associated with the overall perceived stress levels. Thus, COVID-19 pandemia, especially throughout the interval of lockdown, exerted a harmful effect on individuals’ psychological stress levels and emotional eating behaviors, including university students [77].

## 3. Discussion

Comprehensive research was performed in several accurate international literature databases, using multiple relevant keywords and retrieving 44 clinical studies, which evaluated the impact of emotional eating in university students. Several of them (40.9%, n = 18) were retrieved as citations of the initial research papers found in the scientific databases. The above fact shows that, although we used multiple keywords in the most accurate databases to retrieve the relevant clinical studies, several of them were extracted by careful, critical, and time-consuming reading of the incited clinical studies that other researchers discussed in their published articles. This may be ascribed to the fact that emotional eating has been considered as an eating disorder in the last few years. In support of this view, we observed that several studies did not include relevant keywords in the titles and the abstracts such as emotional eating, emotional overeating, emotional undereating, or eating due to emotions. Instead of the above keywords, several older research articles included more general keywords in their titles and/or abstracts such as eating behavior and eating disorder and they indirectly referred to emotional eating in their full text.

Moreover, the vast majority of the retrieved clinical studies (79.9%, n = 35%) have been published in the last five years. This fact shows that research on emotional eating in university students is currently at its first stages. It should also be noted that most of the retrieved clinical surveys included a cross-sectional design (81.8%, n = 36), which cannot support causality effects. In contrast, only a small number of clinical studies had a longitudinal design (9.1%, n = 4). Although most of the cross-sectional clinical studies included an adequate number of university students, there are also some studies with a very low study population. On the other hand, self-reported data were used in the majority of the retrieved clinical studies, which increase the risk of recall biases.

All the currently available clinical studies (except for two clinical studies) used a validated, self-report questionnaire tool. However, there is high heterogeneity concerning the measurement questionnaire tools evaluating emotional eating of the university students. TFEQ was used in most clinical studies (31.8%, n = 14), following EES (18.2%, n = 8), and afterwards DEBQ (13.6%, n = 6) and EEQ (11.4%, n = 5). Among the clinical studies using TFEQ, several versions of this questionnaire tool were used. This heterogeneity concerning the questionnaire tools for evaluating emotional eating may be responsible for certain different results found among the existing clinical studies. This heterogeneity concerning the different questionnaires used also renders the performance of a systematic review quite difficult, as they are not adequate homogenous clinical studies to conduct a meta-analysis of high quality.

A significant number of clinical studies were performed in the USA (20.5%, n = 9), China (13.6%, n = 6), Turkey (11.4%, n = 5), Poland (6.8%, n = 3), and Saoudi Arabia (6.8%, n = 3). One clinical study was performed in Japan, Egypt, Italy, Israel, Thailand, Russia, Korea, Portugal, Malaysia, and Peru, while two clinical studies were performed in France and Ghana. The female/male ratio of the enrolled students was different in each study, with the number of female students being higher than that of male students. Notably, some studies included only female university students (15.9%, n = 7), while two studies included overweight and obese university students. The higher proportion of female university students compared to male university students could be ascribed to the fact that women are more susceptible to suffer from emotional eating in clinical studies performed to the general population. Regarding the above heterogeneities concerning the questionnaire tools, the countries in which the clinical studies were performed render the currently available data not generalized in the general population of the university students. Some contradictory results of the clinical studies may also be ascribed to the above heterogeneity.

A significant number of clinical studies (31.8%, n = 14) showed that BMI was positively related to emotional eating. Nevertheless, in certain clinical studies, this association was weak, while a significant association was not established in one clinical study. Collectively, emotional eating was also shown to be associated with depression, anxiety, and stress of university students. Significant associations between emotional eating and sleep disturbances, physical inactivity, unhealthy dietary habits, female gender, and social media overuse were also reported. It should also be noted that the vast majority of the clinical studies (81.8%, n = 36) have been performed in the last five years, revealing that the scientific community has focused on the evaluation of the emotional eating of the university students only in the last few years. This may be ascribed to the fact that overweight and obesity have gradually increased in children, adolescents, and young adults, taking the characteristic of an epidemic.

Four recent cross-sectional clinical studies examining the effect of the COVID-19 pandemic on the emotional eating of university students were also retrieved. Despite their cross-sectional design, which cannot support causality effects, the COVID-19 pandemic seems to negatively affect the eating behavior of university students, including emotional eating. However, more clinical studies are required for more accurate conclusions to be drawn concerning the impact of COVID-19 pandemia and confinement as well as the long-term impacts of COVID-19 pandemia in relation to the eating behavior of university students.

## 4. Conclusions

To the best of our knowledge, this is the first review that summarizes the impact of emotional eating on university students. In the last few years, there has been an increasing trend of developing emotional eating among university students. The currently available clinical studies supported evidence that emotional eating is associated with female gender, BMI, physical inactivity, depression, anxiety, stress, sleep disturbances, and social media overuse. However, most currently available clinical surveys have a cross-sectional design that cannot establish causality effects with potential risk factors. Thus, there is a strong demand to carry out longitudinal clinical surveys in order for conclusive results to be drawn. Moreover, future clinical studies exploring potential mechanisms explaining the association of emotional eating with psychological factors are highly recommended. Since a significant proportion of university students seem to have emotional eating symptoms, public strategies should be applied to confront this issue. Counseling programs, public health initiatives, and psychological interventions should be applied in the university community in order to efficiently decrease the probability of developing emotional eating behavior in university students.

## Figures and Tables

**Table 1:** Clinical studies assessing the impact of emotional eating in university students.

Study Type	Study Population	EE Assessment *	Basic Results	References
Cross-sectional study	537 Turkish university students	EES	EE was positively related to BMI and body weight. Will, anger, BMI, and body weight were predictors of EE.	Isik et al., 2021 [34]
Cross-sectional study	548 Japanese university underweight and normal-weight students	DEBQ	EE was positively associated with BMI. Amongst obese students, women had higher levels of EE than men.	Ohara et al., 2014 [35]
Cross-sectional study	129 Ghanaian university students	TFEQ	EE was related to BMI concerning males and anxiety, and sleep quality regarding females.	Amoako et al., 2023 [36]
Longitudinal study	1068 Chinese university students	AEBQ	Sex and BMI were identified as significant predictors for harmful EE patterns. Students with emotional over- and under-eating had the highest severity concerning eating disorder symptomatology and psychological distress.	He et al., 2020 [37]
Randomized controlled experimental study	55 Turkish university students	DEBQ	A considerable difference was noted between the pre-test, post-test, and follow-up test BMI of those in the experimental group receiving solution-focused counseling compared to those receiving healthy nutrition training. The short-term solution-focused approach was effective in reducing EE levels.	Saritas et al., 2024 [38]
Longitudinal study	173 USA university students	TFEQ	Baseline EE sub-scales were positively associated with baseline weight, BMI, waist circumference, and fat mass index.	Hootman et al., 2018 [39]
Cross-sectional study	266 Italian university students	EOQ	A psychological pattern of increasing overeating behavior and reduced Self-Directedness in combination with increased Sadness and Anger was found. Males presenting elevated perceived stress at university entrance gained considerably higher body weight during the 2nd semester	Villano et al., 2021 [40]
Cross-sectional study	353 Polish university students	TFEQ	A positive association between EE with EE among females was noted. Social desirability was negatively correlated with uncontrolled eating and EE. Amongst males, physical activity had a main effect on EE.	Kowalkowska et al., 2021 [41]
Cross-sectional study	434 Saoudi Arabia university students	EEQ	EE was related to short healthy eating index score and BMI. Academic major was related to perceived stress and EE.	Sawant et al., 2024 [42]
Cross-sectional study	553 USA university students	AEBQ	Emotional over- and under-eating class had the greatest probability of depression, anxiety, stress, and psychosocial impairment because of eating disorder symptoms as well as decreased psychological flexibility.	Dixit et al., 2023 [43]
Cross-sectional study	272 Saoudi university students	TFEQ	A considerable association of increased social media usage with developing abnormal eating behaviors (EE) was reported.	Shatwan and Alzharani, 2024 [44]
Cross-sectional study	575 Isralian university students	Not validated questionnaire	Students having elevated stress and spending more time on social media were shown more often engaging in EE.	Houminer Klepar et al., 2024 [45]
Cross-sectional study	227 Polish university students	TFEQ	BMI, depression, and impulsiveness scores were higher in participants with a high EE score. Emotional eaters were also characterized by higher scores in cognitive restraint and uncontrolled eating components.	Suwalska et al., 2022 [46]
Validation study	89 Thai university students	TFEQ	Restraint and disinhibition scores were more elevated in females compared to males. Restraint and disinhibition scores were also related to body fat but not to BMI.	Chearskul et al., 2010 [47]
Cross-sectional study	99 African American female university students	REBPQ subscales	Perceived stress was correlated with EE. Stress experience may interact with EE to influence BMI.	Diggins et al., 2015 [48]
Cross-sectional study	1301 Chinese university students	TFEQ	Both EE and depression symptoms were related with laryngopharyngeal reflux symptomatology. Female students exhibited more elevated EE scores compared to males. University students whose major was liberal art, with higher BMI, and with low physical activity levels exhibited increased EE scores.	Liu et al., 2020 [49]
Cross-sectional study	584 Spanish university students	EEQ	Almost 38.6% of the students were categorized as eating very emotionally or eating emotionally, and 37.2% were classified as low emotional eaters. EEQ was weakly, positively associated with BMI in female students.	López-Moreno et al., 2021 [50]
Cross-sectional study	373 Chinese overweight and obese university students	EES	Physical activity considerably influenced self-identity and social anxiety that considerably impacted EE.	Wang et al., 2024 [51]
Cross-sectional study	1000 Korean university students	Not validated questionnaire	Female students were found to tend to eat to release stress as a way to decrease this kind of mood.	Choi, 2020 [52]
Cross-sectional study	424 Chinese university students	DEBQ	More than 3-fold greater probability of negative EE was found among females compared with their male counterparts. Having at least mild depressive symptoms was the only independent parameter related to negative EE amongst males. Amongst females, harmful EE was independently associated with depressive symptoms and mild stress.	Sze et al., 2021 [53]
Cross-sectional study	813 Chinese university students	DEBQ	After adjusting for sex, age, and BMI, sleep quality was identified as a predictor factor of EE. Physical activity levels attenuated the association of sleep quality with EE through depression. Depression was also identified as a crucial predictor of EE amongst students with high physical inactivity.	Zhou et al., 2024 [54]
Longitudinal study	232 USA university students	Emotion and Stress-Related Eating subscale of the (EADES)	At baseline, EE was significantly associated with perceived stress, barriers to and motivators of healthy eating, and avoidance coping. Baseline stress levels were not related to EE one year later.	Dalton et al., 2024 [55]
Cross-sectional study	56 Spanish university students	EEQ	EE and internet overuse were associated with impulsivity, depression, and anxiety. Impulsivity and depressive symptoms were responsible for 45% of the EE variance.	Marchena-Giráldez et al., 2024 [56]
Cross-sectional study	523 USA university students	EES	Perceived stress was positively correlated with EE and negatively correlated with eating self-regulation. A positive association between EE and the consumption of sweets and soft drinks was noted.	Ling et al., 2021 [57]
Exploratory qualitative study	16 USA university students	WREQ	Females recognized stress as the main cause for EE, more often followed by guilt. Males were mainly characterized by unpleasant feelings like monotony or anxiety, turning to food as a distraction.	Bennett et al., 2013 [58]
Cross-sectional study	377 French female university students	EOQ and TFEQ	Half of the students reported intermittent emotional overeating in the last 28 days. A positive association of distress-induced overeating with inability to resist emotional cues, disordered eating symptomatology, and loss of control over food consumption was noted.	Constant et al., 2018 [59]
Exploratory qualitative study	43 USA female university students	TFEQ	Self-assessment of EE in the aggregate was not related to enhanced food consumption in response to stress or harmful emotions.	Klatzkin et al., 2022 [60]
Cross-sectional study	258 Portuguese university students	DEBQ	Utmost all eating behaviors’ dimensions (all except external eating and flexible control in the male group) were considerably ascribed to BMI and psychopathological disorders.	Poínhos et al., 2018 [61]
Cross-sectional study	673 Chinese university students	DEBQ	A positive association of fear of compassion for self and fear of compassion from others with emotional regulation problems, which, in turn, were associated with EE for female students, was noted. For male students, merely a positive association of fear of compassion for self with emotional regulation problems was noted.	Zhang et al., 2021 [62]
Cross-sectional study	300 Polish university students	TFEQ	Students presenting higher BMI, no healthy nutritional habits, reduced levels of physical activity, who underestimated meal size concerning weight and calories, and exhibited enhanced-stress feelings were more probable of developing EE.	Grajek et al., 2022 [63]
Cross-sectional study	850 Turkish university students	TFEQ	An increase in BMI of students was associated with a reduction of 25.6% in the TFEQ scores. The students’ BMI and TFEQ scores depended by their MEQ scores.	Arslan et al., 2022 [64]
Cross-sectional study	372 USA university students	TFEQ	Social jet lag considerably predicted reduced intuitive eating and greater EE. Sleep quality significantly predicted intuitive eating, EE, and loss of control overeating.	Vrabec et al., 2022 [65]
Cross-sectional study	567 female Russian university students	TFEQ	Cognitive eating restraint and uncontrolled eating were associated with the morning subscale. EE was associated with the evening subscale MEQ.	Budkevich et al., 2021 [66]
Cross-sectional study	252 Spanish university students	EEQ	State-anxiety was an identified predictive factor of the emotional eating score and its subscales, and gender was identified as a predictive factor of subscale guilt and the total emotional eating score.	Carlos et al., 2020 [67]
Cross-sectional study	380 Saudi Arabia female university students	EES	Fat intake and educational level were substantially related to emotional eating, and they may be significant predictor factors of EE.	Alharbi et al., 2023 [68]
Cross-sectional study	400 Ghanian university students	EES	A considerable percentage of females compared to males were emotional eaters. Fat consumption was a crucial predictive factor of EE and feelings of enthusiasm.	Intiful et al., 2019 [69]
Cross-sectional study	483 Turkish university students	EEDS	EE was positively associated with mukbang addiction and problematic internet use.	Elkin et al., 2024 [70]
Longitudinal study	132 Malaysian university students	TFEQ	EE scores were substantially different over levels of academic burnout next to 6–8 weeks.	Kristanto et al., 2016 [71]
Cross-sectional study	232 USA university students	EES	Food insecurity was positively associated with EE. The above relationship was higher in male compared to female students.	Frank et al., 2023 [72]
Cross-sectional study	161 Spanish female university students	EEQ	Emotional eaters compared to not emotional eaters showed low food addiction, a considerably enhanced consumption of carbohydrates, fat, and alcohol during the last wave of COVID-19 pandemia.	Díaz-Ureña et al., 2024 [73]
Cross-sectional study	456 Peruvian university students	MEQ**	EE was associated with the overall emotional effect of COVID-19, BMI, depression and anxiety levels, and living with only one parent.	Zeladita-Huaman et al., 2024 [74]
Cross-sectional study	302 French female university students	EOQ	Emotional overeating positively correlated with internal monotony proneness, smoking, attentional impulsivity, lack of ability to avoid emotional cues, and control loss over food consumption and harmfully with age and well-being during COVID-19 lockdown.	Constant et al., 2023 [75]
Cross-sectional study	1000 Turkish university students	EES	EE, eating behavior, and BMI were influenced in students receiving distance education due to social media use throughout the COVID-19 pandemic.	Eser Durmaz et al., 2022 [76]
Cross-sectional study	250 Egyptian university students	EES	COVID-19 pandemia, especially throughout the intervals of lockdown, had a harmful effect on people’s psychological stress and EE behaviors.	Shehata et al., 2023 [77]

* EE: Emotional Eating, EES: Emotional Eating Scale, BMI: Body Mass Index, DBEQ: Dutch Eating Behavior Questionnaire, TFEQ: Three-Factor Eating Questionnaire, AEBQ: Adult Eating Behavior Questionnaire, EOQ: Emotional Overeating Questionnaire, EEQ: Emotional Eater Questionnaire, REBPQ: Revised Eating Behavior Pattern Questionnaire, Emotion and Stress-Related Eating subscale of the Eating and Appraisal Due to Emotions and Stress (EADES), WREQ: Weight Related Eating Questionnaire, MEQ: morningness–eveningness questionnaire, EEDS: Emotional Eating Disorder Scale, MEQ**: Mindful Eating Questionnaire.

## Data Availability

Data are available upon request to the corresponding author.
